# Assessing Tumorigenicity in Stem Cell-Derived Therapeutic Products: A Critical Step in Safeguarding Regenerative Medicine

**DOI:** 10.3390/bioengineering10070857

**Published:** 2023-07-19

**Authors:** Zongjie Wang

**Affiliations:** 1 Department of Biomedical Engineering, McCormick School of Engineering, Northwestern University, Evanston, IL 60208, USA; zongjie.wang@northwestern.edu; 2 Chan Zuckerberg Biohub Chicago, Chicago, IL 60607, USA

**Keywords:** cell therapy, stem cells, tumorigenicity, regenerative medicine

## Abstract

Stem cells hold promise in regenerative medicine due to their ability to proliferate and differentiate into various cell types. However, their self-renewal and multipotency also raise concerns about their tumorigenicity during and post-therapy. Indeed, multiple studies have reported the presence of stem cell-derived tumors in animal models and clinical administrations. Therefore, the assessment of tumorigenicity is crucial in evaluating the safety of stem cell-derived therapeutic products. Ideally, the assessment needs to be performed rapidly, sensitively, cost-effectively, and scalable. This article reviews various approaches for assessing tumorigenicity, including animal models, soft agar culture, PCR, flow cytometry, and microfluidics. Each method has its advantages and limitations. The selection of the assay depends on the specific needs of the study and the stage of development of the stem cell-derived therapeutic product. Combining multiple assays may provide a more comprehensive evaluation of tumorigenicity. Future developments should focus on the optimization and standardization of microfluidics-based methods, as well as the integration of multiple assays into a single platform for efficient and comprehensive evaluation of tumorigenicity.

## 1. Introduction

Stem cells are a unique type of cell with the potential to differentiate into multiple cell types. They possess two fundamental characteristics [1]: the ability to self-renew indefinitely under specific conditions, allowing them to proliferate limitlessly, and the capacity to differentiate into diverse cell types with appropriate biochemical and biophysical stimuli. Under optimal conditions, stem cells have the potential to generate specific cell types endlessly, making them an attractive source of cells for regenerating damaged or aging tissues in vivo [2]. Besides, stem cells are valuable tools for drug discovery [3]. When combined with high-throughput screening technology (e.g., 3D bioprinting [4,5]), stem cells can be utilized to create thousands of mini tissues in the format of organoid [6] or organ-on-a-chip [7], which facilitates comprehensive drug screening [8].

Currently, stem cells can be broadly categorized into two main groups: pluripotent stem cells (PSCs) and somatic stem cells. PSCs are the cells that naturally occur during embryonic development, and they have the potential to differentiate into any of the three germ layers of the embryo: endoderm, mesoderm, and ectoderm [9,10,11]. These layers give rise to various tissues and organs in the body, such as the stomach lining, lungs, muscles, blood, bones, nervous system, and epidermis. PSCs, therefore, hold significant potential for differentiation. However, since PSCs are only found in embryos (and, for this reason, are also known as embryonic stem cells, ESCs), their use is associated with significant ethical considerations that limit the translation of ESC-mediated therapy. In 2006, Shinya Yamanaka and his team demonstrated that somatic cells, such as fibroblasts, could be reprogrammed into a pluripotent state by introducing four specific genes (*MYC*, *OCT3*/*4*, *SOX2*, and *KLF4*, also known as the Yamanaka factors) [12]. This breakthrough gave rise to induced pluripotent stem cells (iPSCs), which provide a straightforward and versatile approach to generating PSCs from individuals for personalized therapy [13,14,15]. Thus far, iPSCs derived from patients have been successfully differentiated into over 100 cell types [16]. Some of these differentiated cells, such as cardiomyocytes [17] and neurons [18] have already been used in clinical trials [19] to treat degenerative diseases, including heart failure [20], spinal cord injury [18], Alzheimer’s disease, and Parkinson’s disease [21].

Somatic or adult stem cells (ASCs) do not pose ethical concerns as PSCs. Examples of ASCs include mesenchymal stem cells (MSCs) in bone marrow and adipose tissues [22], hematopoietic stem cells (HSCs) from umbilical cord blood [23], neural stem cells in nervous systems [24], epithelial stem cells in intestinal crypt [25], and retinal stem cells in retinal periphery [26], and skin stem cells located in the epidermis [27]. The origin of ASCs makes their isolation straightforward. However, ASCs have limited differentiation potential and can only differentiate into a restricted number of cell lineages [22]. For example, MSCs can differentiate into adipocytes [28], osteoblasts [29], chondroblasts [30], neuroectodermal [31,32] and hepatocytes [33]. Therefore, MSCs hold great promise for regenerating specific types of tissues but are not a universal solution for organ regeneration. The translation of ASCs to the clinic varies on a case-by-case basis. While the transplantation of HSCs has been approved by the FDA and is the gold standard for reconstructing the immunity of immunodeficient patients [34], the use of MSCs to treat chronic diseases is still in the early stages of development, with ongoing phase I/II trials [35].

**Figure 1:**
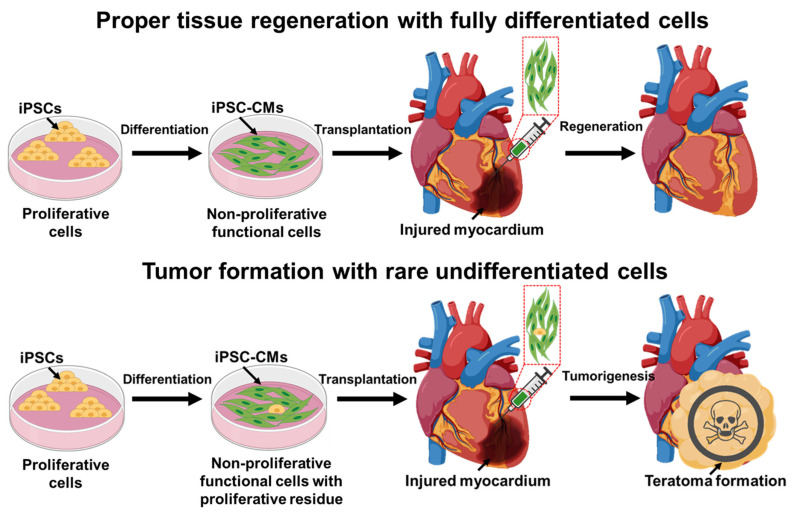
Pluripotent stem cell-derived functional cells for tissue regeneration and associated tumorigenesis risk due to rare undifferentiated stem cells. iPSC: induced pluripotent stem cells. CM: cardiomyocytes.

The use of stem cells as a therapeutic agent for tissue regeneration holds great promise, but it also comes with potential risks, such as graft-versus-host disease [36] and tumorigenicity [37] (Figure 1). The self-renewal properties of stem cells can lead to uncontrolled proliferation and tumorigenicity. On the one hand, undifferentiated PSCs can robustly form teratoma—a type of stem-cell-derived cancer-in animal models [38]—and therefore, PSCs must be sufficiently differentiated into non-pluripotent cell types for therapy. However, it is common that a small number of undifferentiated PSCs will persist in the differentiated cell populations. According to the observation, these rare undifferentiated PSCs presented in the differentiated cell populations can still develop into teratomas [39] or cysts [40], which poses a significant safety concern for PSC-derived therapy. In one case report, a 49-year-old patient with type 2 diabetes received iPSC-derived beta cells as therapeutics and developed a mass with enlarged axillary lymph nodes at the injection site within two months [39]. Most cells in the mass were further confirmed to be OCT3/4 and SOX2 positive, demonstrating their potential origin from PSCs.

On the other hand, the safety risk of MSCs usually does not form tumors, and therefore MSCs are used directly in regenerative therapies in the format of stem cells. However, the tumorigenic risk of MSCs is not zero. In one case report, the intrathecal infusion of MSCs from unreliable sources resulted in glioproliferative lesions in a 66-year-old patient [41], which drew worldwide attention [42,43] and led to an FDA statement in 2017 to enforce proper oversight of stem cell products [44]. Since then, significant efforts have been made to reduce or eliminate the tumorigenicity of stem cell products.

Several studies hypothesized that the tumorigenic risk of stem cells largely comes from remaining undifferentiated populations [45], because the injection of undifferentiated ESCs commonly forms teratoma (Figure 1), a stem-cell-derived tumor, in immunocompromised animals [46]. As a result, researchers have invested a significant amount of effort into developing methods to specifically remove undifferentiated stem cells while keeping the differentiated cell populations viable [47]. For example, Ben-David and co-workers screened 5200 small molecules and identified ESC-specific inhibitors named PluriSIn [48]. PluriSIn can eliminate undifferentiated embryonic stem cells in 24 h of culture, leaving differentiated cardiomyocytes viable [49]. Other studies have developed mitochondria-specific dyes [50] and doxorubicin dosages [51] that can specifically label or selectively kill undifferentiated stem cells in cardiomyocyte populations. However, the effectiveness and toxicity of these treatments on other stem cell populations remain unknown. Moreover, the cost of additional small-molecule treatments is not economical at the manufacturing scale. Therefore, it is now widely recognized that manufacturers should conduct quality checks on differentiated stem cell products to determine whether a specific batch requires additional treatment to reduce the risk of tumorigenicity [52].

This review provides an overview of the various methods utilized to assess the tumorigenic potential of stem cell products. The methods discussed include animal models, soft agar culture, polymerase chain reaction (PCR), flow cytometry, and microfluidics. The review explains the working principles of each approach. It provides a comparative analysis of their performance based on sensitivity and turnaround time, presented in an easy-to-read table format. This review aims to provide readers with a comprehensive understanding of the current assessment methods and facilitate the development of next-generation detection assays in a standardized manner.

## 2. Consideration When Assessing Tumorigenicity

The first question when assessing the tumorigenicity of stem cell products is determining the threshold in terms of cell number and time frame. Indeed, it remains unclear how many cells are required to form tumors within a specific time frame. For cancerous cells, it has been shown that a single cancer stem cell can lead to leukemia relapse [53], so a tumorigenicity assessment must have a single-cell resolution to ensure safety [54]. However, no reports suggest that a single stem cell, whether in undifferentiated or differentiated stages, can form tumor tissues in vivo. The threshold cell number for ESC-derived teratoma formation ranges from about 100 [55] to 10,000 [56] cells per million, far above the range of single cells. This is consistent with the observation that ESCs and iPSCs grow based on colonies/clusters. It is difficult for a single ESC or iPSC to survive and expand. Gropp et al. reported that 10 ESCs spiked in Matrigel have 0% tumorigenicity risk in immunocompromised animals; none of the 30 implanted mice developed teratoma [55]. Therefore, a stem cell tumorigenicity assay does not require single-cell resolution, but it should achieve reasonable sensitivity, for example, 0.001% (equal to 100 cells per million).

In terms of period, most researchers have monitored tumor growth in animals for 10 to 36 weeks [56,57], and the FDA recommends in vivo tumorigenicity monitoring during assay development for 4 to 7 months [58]. There are currently no regulations regarding the length of tumorigenicity assay required for batch-to-batch analysis. However, considering the typical turnaround time for stem cell-derived products is about 1 to 3 months [59,60,61], having a lengthy tumorigenicity assay that requires 4 to 7 months is not ideal. It is, therefore, essential to develop rapid, cost-effective, and highly robust methods for assessing tumorigenicity in manufactured stem cell products with reasonable sensitivity.

## 3. Existing Approaches

### 3.1. Animal Model

**Figure 2:**
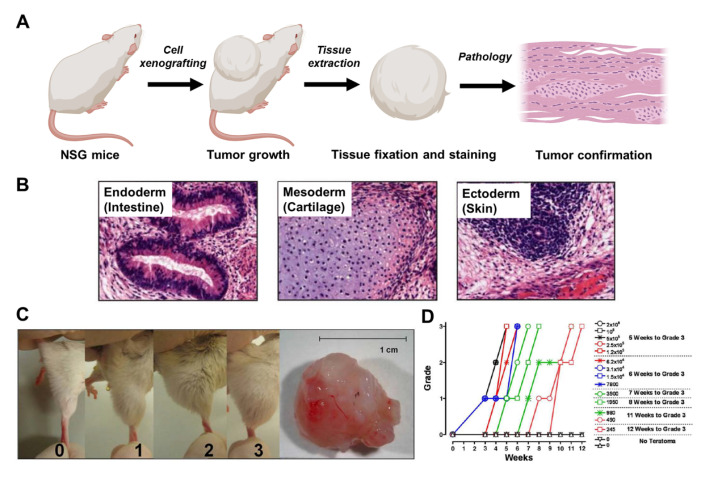
Assessment of tumorigenicity by animal models. (**A**) Workflow of cell xenografting and tissue quantitation. (**B**) Representative H&E images of teratoma containing the tissues derived from three germ layers. (**C**) Development of a simple grading system for visualizing growing tumors. The grading scale is as follows: 0 = no teratoma, 1 = teratoma just visible, 2 = teratoma visible, 3 = large teratoma with a typical size > 1 cm. (**D**) Quantitation of tumorigenicity of rare ESC spiked in non-proliferating feeder cells. Images reprinted from [65] with permission.

Using animal models is still considered the gold standard in assessing the tumorigenicity of certain substrates [62]. In this procedure, stem cell-derived products are xenografted subcutaneously or intramuscularly into immunocompromised mice, commonly NOD-SCID-Gamma (NSG) mice (Figure 2A). The NSG mice are highly immune-deficient, lacking functionality in common immune cell types such as B, T, and NK cells. Therefore, using NSG mice represents the most severe immune suppression condition in human patients who receive HSC transplantation to avoid graft-versus-host disease (GVHD) [63,64].

Injected cells were allowed to grow for up to 7 months before tissue extraction. The extracted tissues were then fixed using formalin and sectioned for immunohistochemistry and immunohistopathology. The cells were usually examined using standard hematoxylin and eosin (H&E) staining for tissue morphology and Ki67 for cell proliferation [66]. In cases where ESC or iPSC-derived cells were used, the tumorigenic cells typically formed teratomas [55], which contain tissue constructs developed from all three germ layers (Figure 2B), including the intestine (Endoderm), cartilage (Mesoderm), and skin (Ectoderm). While histology provides more detailed information, the assessment of tumorigenicity can be simplified to the measurement of engraftment size. For example, Hentze et al. developed a grading system to evaluate the tumor size of engrafted stem cell products (Figure 2C) by measuring the diameter of the hind leg [65]. They reported that this parameter is highly associated with the tumor development of intramuscularly injected cells. Within this system, they quantitatively assessed the tumorigenicity of rare undifferentiated ESCs spiked into feeder cells (Figure 2D). They discovered that even 245 ESC cells spiked into 1 million non-tumorigenic cells are sufficient for tumor initiation. Interestingly, their results showed that the developing teratoma from 245 ESC cells was barely detectable in the first nine weeks, indicating that animal models’ sensitivity at the early stage is insufficient for accurate tumorigenicity assessment. Therefore, animals must be monitored for an extended period to achieve precise quantitation.

Animal models provide a physiologically relevant environment and arguably the best sensitivity for assessing tumorigenicity. However, due to their lengthy workflow, they are better suited as a one-time experiment during product development rather than an assay to be routinely performed during manufacturing, as the stem-cell-derived therapeutics products must be quickly delivered to the patients.

### 3.2. PCR

**Figure 3:**
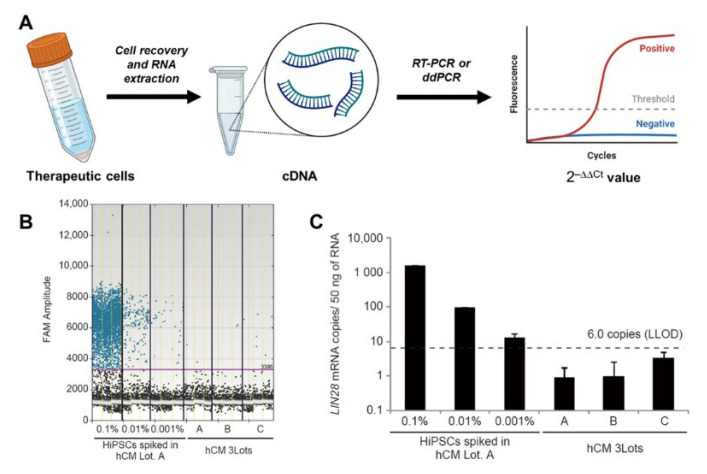
Assessment of tumorigenicity by PCR. (**A**) Workflow of RNA extraction, cDNA synthesis and their use in RT-PCR procedure to determine the gene expression (**B**) Raw data from ddPCR showing the expression of LIN28 mRNA in cardiomyocyte samples with different numbers of spiked-in ESCs. The threshold for positivity was assigned manually. FAM: 6-Carboxyfluorescein. (**C**) Raw data from ddPCR showing the copy numbers of LIN28 mRNA in 50 ng of total RNA. The threshold of the lower limit of detection (LLOD) is determined manually. Images reprinted from [67] with permission.

The polymerase chain reaction is widely used to generate many copies of a specific DNA sample, enabling sensitive quantitation of rare DNA or RNA. More specifically, researchers can use real-time PCR (RT-PCR) and digital droplet PCR (ddPCR) to quantify the expression level of stemness and/or tumorigenicity-related genes. In brief, to perform PCR, bulk cell pellets are collected after centrifugation (Figure 3A), and total RNA is extracted. Complementary DNA (cDNA) is synthesized using reverse transcriptase enzyme and then analyzed via RT-PCR or ddPCR with a standard 40-cycle loop. The 2^−∆∆Ct^ method is used for RT-PCR, while the percentage of positive droplets is used for ddPCR to calculate the expression level of a specific DNA or gene. The entire process, from RNA extraction to results, takes approximately 2–3 h, making it much more practical than animal models.

Using RT-PCR and ddPCR, the ultrasensitive detection of DNA mutations in mammalian cells as low as 0.002% variant allele frequency has been reported [67]. Considering the sensitivity and rapidity of PCR, significant efforts have been made to optimize PCR for the quantitation of rare tumorigenic cells to assess tumorigenicity. One such approach is to use the distinct expression of pluripotent genes in undifferentiated ESCs compared to their differentiated counterparts. For example, Kuroda and colleagues proposed using *LIN28*, a pluripotency [68] and cancer stem cell gene [69], for the detection of undifferentiated ESCs in differentiated cardiomyocytes [70]. By carefully optimizing the threshold for a positive result (based on the fluorescent amplitude of 6-Carboxyfluorescein (FAM), Figure 3B), they reported a LLOD of 0.001% (Figure 3C), demonstrating the great potential of ddPCR as a rapid approach for tumorigenicity assessment.

However, the use of *LIN28* as a sole marker for tumorigenicity remains to be carefully validated, as the expression of *LIN28* in other cell types, such as human brain cells (see https://www.proteinatlas.org/ENSG00000131914-LIN28A accessed on 7 July 2023), may lead to high background and decrease the sensitivity of the assay. Moreover, a recent article suggests that the overexpression of *LIN28* in other cell types (e.g., neural progenitor cells [71]) is not associated with tumor formation, which calls into question the direct connection of *LIN28* expression with tumorigenic phenotypes. Therefore, this gives a rationale to conduct a comprehensive screening of tumorigenicity-associated genes to identify more reliable target genes for PCR analysis. In 2023, Lemmens et al. conducted the first comprehensive screening to detect hallmark genes of undifferentiated iPSCs in iPSC-derived CMs using bulk RNA sequencing [72]. Top-rated gene candidates include *ESRG*, *CAMKV*, *IDO1*, *CNMD*, *LIDT1*, *LCK*, *VRTN*, *ZSCAN10*, and *LIN28*. The validity of these genes for iPSC detection remains to be explored in other iPSC-derived cells.

In addition to traditional transcriptomic biomarkers, recent studies have reported new alternatives at the RNA level for iPSC detection. For example, Tsujimoto et al. discovered a long non-coding RNA (lncRNA) named *MIR302CHG* for the precise detection of iPSC. They achieved a sensitivity of 0.0001% in differentiated nephron progenitor populations using spiked-in samples [73]. In addition, Chung et al. studied publicly archived datasets of microRNA microarrays and discovered a set of microRNA markers, including miR-302a-5p, miR-302c-3p, miR-302d-5p, miR-518f-5p, and miR-519-3p [74]. These microRNAs are highly expressed in iPSCs derived from peripheral blood mononuclear cells compared to differentiated human lymphocytes. The authors demonstrated that the combination of these microRNAs could detect as few as three undifferentiated iPSCs in 10^6^ iPSC-derived natural killer (iNK) cells, corresponding to a LLOD of 0.0003% [74].

The PCR-based method shows great potential as a quick approach for assessing tumorigenicity. Table 1 summarizes the gene candidates reported so far for detecting undifferentiated ESCs and iPSCs. However, the quality of the reported studies is limited by several factors. Firstly, the subjective determination of the threshold is not well justified, which may require detailed disclosure of the laboratory protocols. Furthermore, the cDNA synthesis steps can introduce biased artifacts through various mechanisms, such as primer-independent cDNA synthesis and template switching [75], which may significantly impact low-purity starting materials and should be avoided. Nevertheless, to prevent potential artifacts and biases in this delicate procedure, additional efforts are necessary for target selection and combination.

### 3.3. Cytometry

To avoid the artifacts that PCR can introduce during cDNA synthesis, researchers evaluate the gene expression level at the protein level. This is why different cytometric tools have been introduced to assess tumorigenicity. Flow cytometry is the most widely used cytometric tool, which employs fluidic and optical systems to identify protein expressions from a bulk preparation at the resolution of single cells [76].

In the cytometric procedure, cells are collected as cell pellets and stained with cocktails of antibodies containing multiple fluorescent dyes. The stained samples are then analyzed using a flow cytometer, which focuses bulk cells into single-cell suspension via a sheath flow. The cells are then passed through multiple lasers and filters to measure their fluorescence intensities at specific wavelengths. These measurements are recorded as FCS files and can be analyzed using specialized software such as FlowJo and CytoBank for quantification. Flow cytometry is a destructive but relatively rapid technique capable of processing up to 15,000 events per second [77,78].

Flow cytometry has shown promise in detecting rare cell populations [79,80,81] for diagnostic purposes. However, the author believes flow cytometry is not ideal for quantifying rare cells in stem cell products. Firstly, the sensitivity of flow cytometry largely depends on the number of events recorded. To achieve a sensitivity of 0.01% with a coefficient of variation of 1%, it is necessary to record at least 1 × 10^8^ events [82]. Recording such many cells destructively results in a significant waste of therapeutic cells, reagents, and time (approximately 1.8 h at a rate of 15,000 events per second). Although there are methods to record fewer events for rare cell quantification, there is a considerable degree of non-linearity and poor sensitivity observed at or below 0.4% rarity [83].

New cytometric systems have recently been proposed to detect rare tumorigenic cells (Figure 3B). These systems utilize microengineering techniques to improve capture performance against rare cell populations, offering better sensitivity and reducing the required input cells. During the procedure, cells are collected as cell pellets and stained with antibodies conjugated with magnetic nanoparticles (MNPs). The labeled cells are then introduced into a microfluidic device sandwiched by magnets. The magnetic field interacts with the MNPs [84], generating a magnetic force that allows cells to overcome the fluidic drag force in the microfluidic channel [85,86]. As a result, target cells labeled with MNPs are selectively trapped in the microfluidic device for quantification. Several studies have consistently reported that MNP-mediated cell trapping/sorting achieves much better sensitivity and lower detection limits than flow cytometry [87,88,89,90].

**Figure 4:**
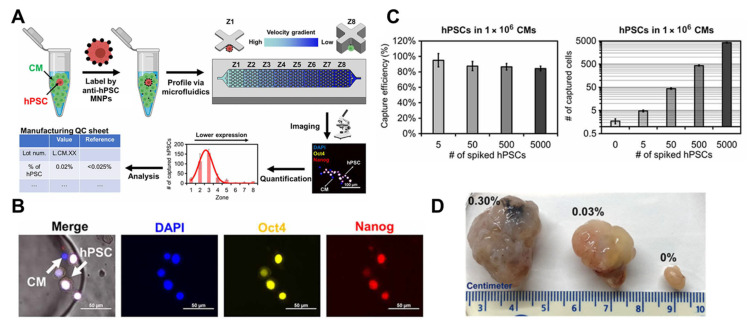
SCQC rapidly identifies undifferentiated hPSCs in differentiated cardiomyocytes (CMs) and definitive endodermal progenitors. (**A**) Overview of the SCQC approach. Cells were magnetically labelled, profiled by a microfluidic capture device, and quantified under a fluorescence microscope. (**B**) Representative microscope images of captured hPSCs on-chip. hPSCs were defined as DAPI^+^, OCT3/4^+^, and NANOG^+^. (**C**) Capture performance of rare hPSC samples spiked in 1 million derived cardiomyocytes. SCQC robustly captured the spike-in rare hPSCs in the 5–5000 cells range. Statistically, a difference was observed between 0 cell and 5 cells, which indicates the limit of detection of SCQC is 0.0005% or better. (**D**) Representative images of the teratomas formed from the cell samples quantified by SCQC. Images reprinted from [57] with permission.

An example of a magnetic cell sorter is the Stem Cell Quantitative Cytometry (SCQC) system (Figure 4) [57]. The SCQC workflow involves three major steps: MNP labeling, microfluidic capture, and microscope quantification (Figure 4A), with a total duration of approximately two hours. In brief, cell mixtures are labeled with MNPs that target TRA-1-60, a protein marker specific to human pluripotent stem cells (hPSCs) on the cellular membrane. Labeled cells are then introduced into the microfluidic device equipped with external magnets and capture pockets. Tumorigenic hPSCs are selectively captured on the device, while other differentiated phenotypes are flushed out to the reservoir. The captured cells are then stained with other pluripotency markers, including *OCT4* [91] and *NANOG* [92], for confirmation (Figure 4B). Finally, the captured and stained cells are quantified under the fluorescence microscope, and the percentage of rare cells is calculated by normalizing to the number of input cells. The SCQC system achieves remarkable sensitivity (0.0005%, 5 cells) with a low input cell number (1 million cells, Figure 4C), demonstrating robust discrimination of undifferentiated hPSCs against cardiomyocytes and definitive endodermal progenitors. The outcomes of SCQC match well with the independently performed teratoma formation results from animal models (Figure 4D).

While SCQC offers a sensitive and low-input solution for tumorigenicity assessment, it is essential to note that the system relies on specific markers or combinations of markers for detection [93,94], similar to PCR. As with any marker-based approach, there is a risk of marker specificity issues. Furthermore, the running protocol for these systems may not be readily available, and it may require significant effort for non-experts to standardize the protocol [95]. Table 2 summarizes the protein biomarkers reported so far for detecting undifferentiated ESCs and iPSCs.

### 3.4. Soft Agar

The PCR and cytometric methods mentioned above rely heavily on specific genes or proteins expressed only in tumorigenic cells and, as a result, lack generalizability. To overcome this limitation, researchers have developed a more generalized assay to assess tumorigenicity at the phenotypic level without labeling.

**Figure 5:**
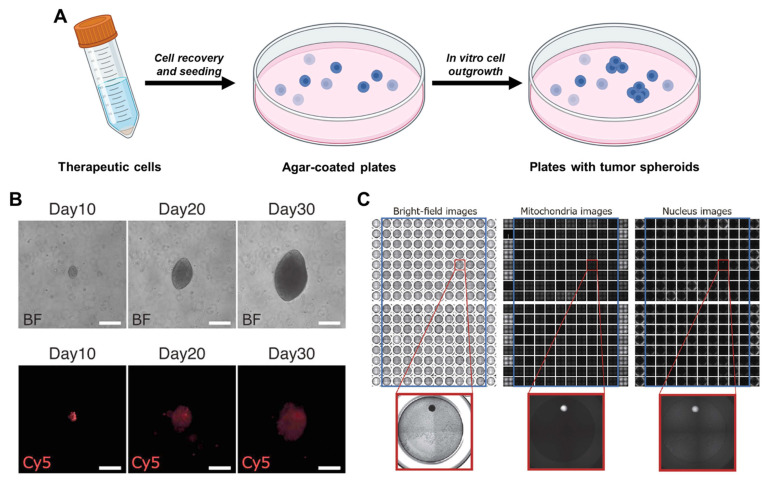
Assessment of tumorigenicity by soft-agar assay. (**A**) Workflow of colony-based assessment of rare tumorigenic cells. (**B**) Representative images of Cy-5 labeled HeLa cells spiked in human mesenchymal stem cells. Due to its tumorigenic nature, HeLa cells form significantly bigger colonies over 30 days’ culture. (**C**) Images of automated detection of the colony formed by the single HeLa cells spiked in human mesenchymal stem cells. The mitochondria images were achieved by staining the wells with MitoTracker. The nucleus images were captured by staining the wells with Hoechst 33342. Images reprinted from [99] with permission.

The soft agar assay, also known as the colony formation assay (Figure 5), is a label-free method for assessing tumorigenicity. This assay is based on the fact that tumorigenic cells exhibit unlimited proliferation and a high level of invasiveness, allowing them to form large colonies from a single cell in an anchorage-independent manner. Compared to non-tumorigenic cells, the colonies formed by tumorigenic cells are larger and more invasive. Because of its high-throughput and label-free nature, the soft agar assay has been popular in the in vitro assessment of tumorigenicity for almost half a century [97,98].

The execution of soft agar assay involves dissociating cells into single-cell suspensions, mixing them with agarose gel, and seeding them onto a culture plate (Figure 5A). The medium is renewed regularly for a few weeks until the growth of tumorigenic cells becomes apparent. Kusakawa et al. used this assay to detect rare HeLa cancer cells spiked into non-tumorigenic human mesenchymal stem cells [99]. They confirmed that HeLa cells could form large colonies over the course of 30 days (Figure 5B) and developed a strategy to automatically quantify the number of large colonies using bright-field, mitochondria, and nucleus images (Figure 5C). They validated their algorithm by detecting a single HeLa cell in a background of 1 million human mesenchymal stem cells, which corresponds to a limit of detection of 0.0001%. Watanabe et al. combined the soft agar assay with magnetic cell sorting and achieved a sensitivity of 0.00002% when detecting undifferentiated ESCs in human mesenchymal stem cells [96]. In addition to assessing the tumorigenicity of stem cell-derived products, the soft agar assay has been used to evaluate the tumorigenicity of gene-edited cells [100] and the efficacy of drugs against tumor-initiating cells [101].

The traditional agar assay has a known limitation: it uses dense, hard-to-degrade biomaterials such as agar as a scaffold for cell growth. This can limit the speed of cell growth. In a study by Kusakawa et al. [99], it took approximately 30 days for highly invasive HeLa cells to form large colonies that were easily detectable under the microscope. A less invasive phenotype is expected to require an even longer period of culture. Moreover, soft agar may not provide sufficient microenvironmental cues to form a stem cell niche, potentially leading to altered phenotypes in growing colonies of ESCs. Although matrix-free culture has been used to improve the turnaround time of traditional agar assays [98,102], the soft agar assay remains time-consuming, taking two weeks or more to yield results, compared to PCR and cytometry. Considering the typical turnaround time for cell therapy, the prolonged soft agar assay should be avoided as a front-line approach for tumorigenicity assessment.

In summary, there is currently no universal solution for assessing tumorigenicity. There is a trade-off between marker dependency, assay time, and sensitivity. The major limitation of the animal model and soft agar assays is the long time for the final results (>1 month), which is incompatible with the demand to generate stem-cell-derived products for rapid delivery and therapeutics. The major limitation of the PCR and cytometry is the reliability of the selected biomarkers, as the gene candidates that clearly and exclusively define undifferentiated ESCs and iPSCs are not comprehensively studied. The characteristics of the assays discussed above are summarized in Table 3 for comparison purposes.

## 4. Outlook and Conclusions

The issue of tumorigenicity in stem cell-derived products has gained significant attention and has been extensively discussed in recent publications [52,103,104]. To address emerging safety concerns, several non-profit organizations, including Cell Therapy-Tracking, Circulation and Safety (CT-TRACS) at the Health and Environmental Sciences Institute (HESI), Non-Clinical Safety Evaluation of Pluripotent Stem Cell-derived Products (CoNCEPT) at the Forum for Innovative Regenerative Medicine (FIRM), and Multisite Evaluation Study on Analytical Methods for Non-clinical Safety Assessment of Hu-man-Derived Regenerative Medical Products (MEASURE), have been formed. In collaboration with academic researchers, these initiatives have published a viewpoint outlining the current challenges in tumorigenicity assessment and proposing a roadmap for the future, with a focus on clinical translation [104]. The initiatives have identified critical points to be considered during large-scale manufacturing and patient administration, such as managing risks related to cell therapy product properties, patient backgrounds, and regulatory guidelines. Due to space constraints, the detailed coverage of these points will not be repeated in this review. Interested readers can access these valuable discussions and ideas online for free [104].

This section is aimed to address three fundamental challenges that must be tackled even before large-scale translation: (1) determining clinically relevant sensitivity, (2) investigating the threshold of rare cells for tumor formation, and (3) conducting extended validation using real-world samples.

The goal of having a sensitive assay is to eliminate the risk of unwanted tumor formation after regenerative therapy rather than achieving a limit of detection that is irrelevant to actual applications. Therefore, it is crucial to determine the clinically relevant requirement for sensitivity [71]. Unlike other pathogens, it remains unclear what level of sensitivity is necessary for tumorigenicity assessment in stem cell-derived products. Using xenografts has established the threshold for human ESCs to grow in mice. Two independent observations have shown that injecting 0.025% ESCs into fibroblasts [65] or cardiomyocytes [57] results in almost 100% teratoma formation in immunocompromised animals. Therefore, the assay may need a detection limit of 0.025% or better. However, using immunocompromised mice for xenografts is still different from the situation in patients. Thus, the exact range of required sensitivity for human applications may need to be determined empirically in the future.

At this moment, the animal model is the best practice for determining the required sensitivity. Researchers can spike different numbers of rare tumorigenic cells into a background of derived cells and xenograft the cell mixtures into animals for comparison. Subsequently, a curve regarding tumorigenicity and rare cell concentration can be derived to define the in vivo threshold for tumor formation. Although this experiment sounds straightforward, it is complicated and may require a global effort. Preliminary studies indicate that several factors, such as the inherent properties of the cell line [46], the type of feeders used [65], the mouse genotypes and immunity [105], the location of xenografting [106], and the dissociation methods used for preparing the injection [65], all strongly impact the possibility of tumor formation. Therefore, the experimental designer must consider conditions that closely mimic clinical administration and execute xenografting accordingly. Additionally, every stem cell product may require an empirical determination of its threshold of tumor formation. Manufacturers can use this threshold as a guideline to develop or select strategies for in vitro tumorigenicity assessment.

Also, assessing tumorigenicity using spike-in samples and xenografting raises concerns about how well these methods recapitulate the clinical situation. For example, spiking in cancer cell lines like HeLa does not accurately represent the biological reality of regenerative therapy. Even PSCs spiked into derived cell products cannot fully represent the undifferentiated residual pluripotent stem cells. Gene expression microarray data from isolated cardiac progenitor cells with undifferentiated residual pluripotent stem cells have shown that these cells have a distinct gene expression profile [57], with significant variation in genes such as *SLC25A1* and *EGLN1* compared to regular PSCs. This unique gene expression profile partially explains why undifferentiated residual pluripotent stem cells are resistant to prolonged differentiation procedures and have stronger pluripotency. As a result, well-optimized assays and tumor thresholds may not be transferable to the case of actual tumorigenic cells given their distinct phenotypes. To better understand the difference between regular cells and rare tumorigenic cells, researchers may need to use single-cell or clonal-specific analysis.

Considering the issues discussed above, the assessment of tumorigenicity still has a long way to go before it can be regarded as mature. It is crucial to understand the phenotype of real rare cells and conduct a comprehensive study on the minimum cell percentage that can initiate tumor formation. Addressing these two fundamental questions will enable researchers to better define their requirements for in vitro tumorigenicity assessment. In addition to the manual efforts, the proper use of advanced technologies, such as automated robotics [107,108], sensitive biosensors [109,110], and machine intelligence [111,112,113], may also help the academia and industry to reach a global consensus on tumorigenicity assessment.

In summary, stem cell therapy holds great promise for treating many diseases, but it must adhere to the principles of bioethics, which include the maxim “First, do no harm”. As healthcare professionals, it is essential to consider and evaluate the potential harm that any treatment may cause. The tumorigenicity inherent in stem cell therapy has received significant attention, and multiple assays have been developed to detect, trace, and eliminate the risk of tumorigenicity associated with stem cell products. Although these approaches are still in their early stages and require further evaluation, they represent our commitment to developing safe and effective stem cell products for clinical use. With the collaboration of biologists, bioengineers, veterinarians, and clinicians, a day may be envisioned when high-quality stem cell products transforming the course of tissue degeneration can be confidently produced.

## Figures and Tables

**Table 1:** List of gene candidates for the detection of undifferentiated ESCs and iPSCs.

Marker	Approach of Discovery	LLOD	References
*LIN28* or *LIN28A*	RT-PCR on a panel of 7 common iPSC markers	0.001%	[70]
*ESRG*, *CAMKV*, *IDO1*, *CNMD*, *LIDT1*, *LCK*, *VRTN*, *ZSCAN10*	Bulk RNA-sequencing	0.001–0.1%	[72]
*MIR302CHG*	Bulk RNA-sequencing	0.0001%	[73]
microRNA 300 and 500 families	miRNA microarray	0.0003%	[74]

**Table 2:** List of protein candidates for the detection of undifferentiated ESCs and iPSCs.

Marker	Approach of Discovery	LLOD	References
SSEA-4	Flow cytometry on a panel of 11 common iPSC markers	Not reported	[57,96]
TRA-1-60	Flow cytometry on a panel of 11 common iPSC markers	<0.0005%	[57]
EpCAM (CD326)	Flow cytometry on a panel of 11 common iPSC markers	Not reported	[57,96]
Tetramethylrhodamine methyl ester perchlorate (TMRM)	Rationale analysis	<0.1%	[50]

**Table 3:** Comparison of the approaches to assess tumorigenicity in stem cell-derived products.

	Animal Model	PCR	Cytometry	Soft Agar
Principle	In vivo cell outgrowth	mRNA expression	Protein expression	In vitro cell outgrowth
Sensitivity	N/A	0.001%	0.0005%	0.0001%
Assay time	1–7 months	3 h	1.5–2 h	2–6 weeks
Bias	Low	High (due to cDNA synthesis)	Low	Medium (may discriminate suspended cells)
Major limitations	Long assay time Non-scalable Labor-intensive	Subjective cutoff Marker-mediated	Marker-mediated Non-standard equipment	Long assay time Labor-intensive
References	[55,65]	[70]	[57]	[96,99]

## Data Availability

Not applicable.
